# Correction: Sequential treatment of metastatic renal cell carcinoma patients after first-line vascular endothelial growth factor targeted therapy in a real-world setting: epidemiologic, noninterventional, retrospective–prospective cohort multicentre study

**DOI:** 10.1007/s00432-025-06161-6

**Published:** 2025-03-26

**Authors:** Alvydas Cesas, Vincas Urbonas, Skaiste Tulyte, Rasa Janciauskiene, Sigita Liutkauskiene, Ingrida Grabauskyte, Ignas Gaidamavicius

**Affiliations:** 1 Klaipeda University Hospital, Klaipeda, Lithuania; 2 National Cancer Institute, Vilnius, Lithuania; 3 Clinic of Internal Diseases, Family Medicine and Oncology, Faculty of Medicine, Institute of Clinical Medicine, Vilnius University, Vilnius, Lithuania; 4 Oncology and Hematology Department, Oncology Institute, Lithuanian University of Health Sciences, Kaunas, Lithuania; 5 Department of Physics, Mathematics and Biophysics, Lithuanian University of Health Sciences, Kaunas, Lithuania; 6 UAB “Guruma”, Kaunas, Lithuania

**Correction to: Journal of Cancer Research and Clinical Oncology (2023) 149:6979-6988**

10.1007/s00432-023-04645-x

In this article, the affiliation details for the author Rasa Janciauskiene were incorrectly given as ‘Clinic of Oncology and Hematology, Institute of Oncology, Lithuanian University of Health Sciences Hospital of Lithuania, Kaunas Clinics Hospital, Kaunas, Lithuania’ but should have been ‘Oncology and Hematology Department, Oncology Institute, Lithuanian University of Health Sciences, Kaunas, Lithuania’.

The affiliation details for the author Sigita Liutkauskiene were incorrectly given as ‘Hospital of Oncology, Affiliate Hospital of Lithuanian University of Health Sciences, Kaunas, Lithuania’ but should have been ‘Oncology and Hematology Department, Oncology Institute, Lithuanian University of Health Sciences, Kaunas, Lithuania’.

The original article has been corrected.

